# Directed Organ Donation After Euthanasia

**DOI:** 10.3389/ti.2023.11259

**Published:** 2023-05-30

**Authors:** Nathalie van Dijk, David Shaw, Sam Shemie, Kim Wiebe, Walther van Mook, Jan Bollen

**Affiliations:** ^1^ Department of Intensive Care Medicine, Maastricht University Medical Center+, Maastricht, Netherlands; ^2^ Care and Public Health Research Institute, Maastricht University, Maastricht, Netherlands; ^3^ Institute for Biomedical Ethics, University of Basel, Basel, Switzerland; ^4^ Division of Critical Care, Montreal Children’s Hospital, McGill University Health Centre, Montreal, QC, Canada; ^5^ Canadian Blood Services, Ottawa, ON, Canada; ^6^ Medical Assistance in Dying, Shared Health Services, Winnipeg, MB, Canada; ^7^ Department of Anesthesiology, Pain and Palliative Medicine, Radboud University Medical Center, Nijmegen, Netherlands

**Keywords:** recipient selection, end of life, deceased donation, euthanasia, directed donation

## Abstract

Organ donation after euthanasia is performed in Belgium, the Netherlands, Canada and Spain. Directed deceased organ donation is currently possible under strict conditions in a limited number of countries, while it is currently not possible to opt for directed donation following euthanasia. While organ donation after euthanasia is a deceased donation procedure, directed organ donation after euthanasia could be seen as a deceased donation procedure with a living donation consent process. Therefore, directed organ donation after euthanasia is feasible on medical and ethical grounds. Strict safeguards should be in place, including the requirement of a pre-existing familial or personal relationship with the proposed recipient, without any evidence of coercion or financial gain.

## Introduction

The majority of deceased organ donations occurs in patients who were comatose and could not provide first person consent. Physicians rely on surrogate decisions and/or their donor registration. Most countries do not allow “directed deceased donation,” wherein it is possible to choose a specific recipient. Organs from a deceased donor are allocated to those who most urgently need it on the transplant waiting list. This differs from living donors who can donate their organ to specified recipients, most often a relative.

Organ donation after euthanasia is being performed in Belgium and the Netherlands for several years [1]. Research based on Belgian euthanasia data demonstrated that 10% of patients who undergo euthanasia might be medically eligible for organ donation [2]. The majority of euthanasia patients however suffer from malignancy, which makes them unsuitable for organ donation [2,3]. Canada legalized euthanasia (which is referred to as “medical assistance in dying” (MAID)) in 2016, and after multiple patient-initiated requests, implemented organ donation after euthanasia in accordance with national guidelines [4,5]. In 2021, this combined procedure also became possible in Spain.

Euthanasia requires the administration of intravenous drugs by a physician, in contrast with (physician) assisted suicide, where a patient can take a lethal medicine themselves [6]. Euthanasia and assisted suicide are currently subject of debate in a growing number of countries.

Patients who choose euthanasia are conscious and competent, which makes them capable of making a well informed decision about organ donation after euthanasia, but which could also allow them to choose a specific recipient for their organ(s). Directed donation after euthanasia is generally not possible, either because a country does not allow euthanasia, or because directed deceased donation is not allowed [7]. According to Cronin and Price, directed and conditional donations provide immediate and evident challenges to the traditional construct of altruistic donation and impartial (equitable) allocation [8].

This article addresses the observation that an increasing number of countries allow euthanasia, and how organ donation organizations can respond to patient requests for directed donation after euthanasia. The ethical aspects of autonomy, vulnerability, distribution of resources and avoiding organ trade are discussed more in detail, since these seem to be the most significant threshold for allowing directed organ donation after euthanasia. Next, we propose a set of criteria under which it would be appropriate to proceed with directed donation following euthanasia.

### Can Directed Organ Donation After Euthanasia be Legal?

Uniform to all euthanasia laws, which vary by jurisdiction, a patient can undergo euthanasia only if they are suffering from a grievous and irremediable condition, if they have intolerable suffering that cannot be alleviated under conditions acceptable to the patient, if they are mentally competent, and if the request has also been evaluated and deemed eligible by a second independent physician.

The Organ Donation Acts in Belgium and the Netherlands state that in a deceased donation procedure, neither the donor nor his relatives are allowed to choose a recipient. Allocation is legally performed through Eurotransplant, an organization that allocates donated organs for eight European countries, depending on urgency and compatibility. In Canada, policy and practice regarding patient initiated requests for deceased directed donation varies per province/territory [9].

The current Canadian policy on donation after euthanasia states that directed donation should not be offered or encouraged [4]. If a patient insists on directed donation, the request should be carefully considered on a case-by-case basis. This has occurred at least once, although the directed donation was not possible as the recipient did not have a compatible blood type [10,11]. Directed organ donation after euthanasia is thus very likely to happen in Canada, as the current legislation does not prohibit it.

Belgium and the Netherlands allow living kidney donors to choose a specific recipient, but directed donation after euthanasia is not allowed because it is legally a deceased donation. We feel that directed organ donation after euthanasia is actually a deceased donation procedure with a living donation consent process.

In the United States, India and the United Kingdom, directed deceased donation (without euthanasia) is allowed under strict conditions [12–14]. As an example, the UK policy requires that the request for the allocation of an organ to a specific recipient should be to a relative or friend of long standing, while no other patients are in urgent clinical need of the organ, that the specific recipient is on the transplant waiting list or could be considered to be placed on the waiting list, and that in life, the deceased had indicated a decision to donate to a specific recipient in need of an organ, or, in the absence of that indication, that the family of the deceased expresses such a decision. However, the consent for directed organ donation is not allowed to be conditional, so if not all requirements for directed donation can be met, organ donation should proceed to other recipients.

### What are the Medical Considerations?

From a medical point of view, a deceased directed donation procedure could result in better compatibility between the donor and the recipient, due to better human leukocyte antigen matching, assuming the donor and recipient are commonly relatives [15]. Chances of a successful transplantation would therefore be higher compared to an unrelated donation after allocation by the transplant organization, based on the transplant waiting list. Research demonstrates that lungs, kidneys and livers transplanted following organ donation after euthanasia function adequately [16–18]. Recently, the Netherlands started heart donation following euthanasia as well, which will also have a significant impact on the transplant waiting lists—even though this is not the primary goal [19].

### Would it be Ethical to Allow Directed Organ Donation After Euthanasia?

There are several ethical aspects that need to be addressed in the context of this combined procedure.

#### Vulnerability and Autonomy

As stated by Case et al., “patients wishing to donate organs as part of the euthanasia process are a population that might be considered vulnerable and in need of protection given perceived threats to their autonomy” [10]. The possibility of directed donation after euthanasia gives patients the opportunity to help close family or friends after death by providing an organ for transplantation. In this sense, euthanasia enables patients to benefit other patients, in line with the principle of beneficence. However, this potential benefit does complicate the consent process for both euthanasia and donation, as there is a potential risk that the possibility of donating to a relative or friend might compromise or contaminate the consent process for euthanasia. The principle of respect for autonomy requires that any decision to engage in euthanasia (with or without organ donation, whether directed or not) is voluntary, and free from any potentially coercive influences. For example, a patient who is terminally ill might either choose to die sooner through euthanasia as waiting for a natural death might deny their relative the organ. In addition, the donor’s commitment, once made, may influence the ability to change their mind about continuing euthanasia, because of a desire not to “let down” the intended recipient.

However, these concerns should be addressed carefully. It is recommended that the patients request organ donation after euthanasia themselves, and, the medical team must attempt to establish that such factors do not play a role in the decision to choose euthanasia, acknowledging that this may be challenging. Furthermore, it is always made clear to patients that they can change their mind about euthanasia or donation at any point. This is also important in view of the principle of non-maleficence. While voluntary consent to euthanasia and directed donation are not in principle incompatible, the safeguards mentioned here are reliant upon full honesty from the patient, and it may be difficult in practice to ensure that the decisions being made are fully autonomous. Even if the patient is fully honest, it might be hard for the patient themselves to be entirely sure whether the wish to donate influences the decision to seek euthanasia. Even though the consent procedures for euthanasia and donation are separate, in the patient’s mind they may be very closely linked in a way that could make full voluntariness challenging.

Generally, both procedures should be kept as separate as possible, and it should also be investigated whether any reciprocal obligation would arise with the recipient or his relatives. The recipient of the organ, or members of their social network might also feel more social pressure and obligation to the donor’s family—which is a negative consequence of the donor not being anonymous, as is the case in directed living donation as well.

At the same time, one could argue that, the principle of autonomy means that one should be able to decide to choose euthanasia in order to donate an organ to a relative. However, given that one can only pursue euthanasia if one is suffering hopelessly and unbearably, one is already in a vulnerable position, and society will expect physicians to protect these patients. If one would allow euthanasia because of a wish to donate, even if all other due diligence requirements are fulfilled, this might currently have a negative impact on the public view on organ donation in general—which should certainly be avoided.

Organ donation after euthanasia based on psychiatric suffering is already possible in Belgium and the Netherlands. In the Netherlands, about one in four cases of organ donation after euthanasia was the result of psychiatric suffering, and 115 (1.3%) of Dutch euthanasia cases in 2022 was due to psychiatric suffering [20]. Euthanasia because of mental illness is not possible in Canada until March 17, 2024.

#### Avoiding Organ Trade

There is also a risk of commercial trading in organs [21,22]. The patient who is about to donate his organs after euthanasia might have found a request on social media from a patient who is willing to pay for an organ. The treating physician and the consulted independent physician (as required by the euthanasia procedure), and perhaps the organ donation coordinator, should investigate the wish of the patient to donate to a specific person who is not a family member or pre-existing friend. However, the same criticism applies to cases of directed living donation and this is not a categorical objection to directed donation after euthanasia.

#### Resources

Deceased directed donation could be seen as involving unfair distribution of scarce resources, since the transplant waiting lists are bypassed, and someone on that list might be in higher need of an organ than the patient who actually receives it. However, this is not any different from living donation directed to a specific individual, and someone lower on the list could receive an organ more quickly as it will remove someone from the transplant list. It is possible that directed donation after euthanasia would exacerbate existing socio-economic inequalities by benefitting both donors and recipients with large social networks, greater social media skills and better socioeconomic positions: donors are more likely to be able to identify someone in need, and those in need with large networks are more likely to be able to find a donor [23]. Again, the same issues of justice also affect directed living donation, where they are not seen as fundamental reasons to prevent the practice.

The same applies to the context of directed donations to a specific group or class, without specifying a particular individual. For example, if a member of an equity deserving group (e.g., indigenous person) undergoes euthanasia, they may wish the organ to be donated back to their community without having a specific individual in mind. One can envision many populations who are disadvantaged by structural inequities in the system that may wish to repair this inequity through directed donation to a class. Although very understandable, this seems to go against the principle of justice which is an important aspect of organ donation policies.

Scientific literature has discussed the effectiveness of directed donations in achieving specific goals, such as reducing poverty or improving health outcomes in specific populations [24,25].

In terms of the principle of beneficence, facilitating the patient’s last wish through allowing them to donate their organs after euthanasia benefits both the patient and the recipients. There does not seem to be a relevant difference whether an organ is donated through standard allocation or through directed organ donation after euthanasia, except inasmuch as helping a known recipient may benefit the patient more than helping a stranger. If one would refuse directed organ donation after euthanasia, there is a risk that the potential donor will not choose organ donation at all, consequently also affecting other patients that would receive an organ.

## Discussion

Directed organ donation after euthanasia may be legally, medically and ethically acceptable. It is an increasingly timely issue to be addressed as more and more jurisdictions enact legislation permitting euthanasia.

If directed organ donation after euthanasia is not possible, two theoretical alternatives exist for a patient who will undergo euthanasia and wishes to donate to a specific recipient. The patient could request a directed living donation a few days before undergoing euthanasia. The majority of patients who undergo organ donation after euthanasia suffer from a neurodegenerative disease which poses a high risk for anesthesia and surgery [2]. A living donation procedure could thus potentially cause death or influence the patient’s quality of life in his last days. A patient who is already suffering unbearably is likely not interested in spending his last days in the hospital to undergo surgery before undergoing euthanasia.

The second theoretical alternative would be “organ donation euthanasia”: anesthetizing the patient and donating his organs [26]. However, such hypothetical “death by donation” procedure is legally considered a living donation procedure, a procedure during which the donor is legally not allowed to be harmed, and which is therefore illegal. To circumvent this issue, a patient who fulfills all criteria for euthanasia might be anesthetized to donate one kidney, as a living directed donation procedure, immediately followed by administration of the euthanasia drugs by his own physician. This would then result in death, which would make it possible to procure all other organs (non-directed) following the no touch period, still respecting the dead donor rule. However, this is currently still a hypothetical situation that would be in contrast with the requirement that a patient reaffirms their euthanasia request immediately before the euthanasia drugs are administered. In the Netherlands this was also deemed to be an issue in procedures where a patient is sedated at home before being transported to the hospital where euthanasia and organ donation are performed. However, euthanasia review committees have judged that the euthanasia due diligence requirements were still fulfilled in these cases [27].

Opponents of euthanasia worry about coercion, which was also one of the main criticisms in the discussion during the referendum on the End of Life Choice Act in New Zealand. If directed organ donation after euthanasia would be available, and if a terminally ill patient’s relative would be in need of an organ, this was considered to potentially lead to an enormous pressure on the patient to choose this combined procedure. While it would also be unjust not to allow this patient to donate to his relative, safeguards are essential to investigate the social situation and to avoid any coercion. The patient should always be able to refrain from organ donation after euthanasia or from euthanasia itself.

Given all of the above arguments and based on what we can learn from areas that currently allow for directed deceased donation, directed organ donation after euthanasia could be permitted under certain circumstances, subject to rigorous safeguards.

These should include:

• the request for directed organ donation after euthanasia is a voluntary request made by the conscious donor who is about to undergo this combined procedure
• the request should be donor initiated without any evidence of coercion or financial gain
• the donor should have a pre-existing familial or personal relationship with the proposed recipient, to avoid potential commercial trading and distrust from the public
• the intended recipient is on the waiting list or meets the listing criteria, and the donor organ is medically compatible for the intended recipient
• the donor should be able to refrain from either procedure until the very last minute.

In the requirements that are applied in the UK (where euthanasia is not allowed), as mentioned above, there is the requirement of unconditionality in case there are other patients in urgent clinical need of the organ. However, this seems in essence unenforceable, and it opposes the patient’s autonomy while the latter principle is at the center of the organ donation after euthanasia procedure. If the donor would be informed about another patient in more clinical need, they might decide to postpone dying to still be able to perform a directed donation.

In Canada, introducing directed organ donation after euthanasia would only require a change in guidance, while in Belgium and the Netherlands, the laws on organ donation would need to be adjusted. Eurotransplant currently does not have a policy on directed deceased donation. Practically, allocation to the specific recipient(s) can be processed just as this would be done for living directed donation. Next to the directed donation of one organ, the recipients of other donated organs can still be selected based on the transplant waiting lists–while directed donation of more than one organ is possible as well. We concede that the proposed procedure of directed organ donation after euthanasia will be very rare, but nevertheless we need to discuss this topic and potentially adjust the law, since it would seem unjust if a patient who wants to donate to a specific relative following euthanasia does not get this chance because of legal requirements.

## Conclusion

Directed organ donation after euthanasia is medically, legally and ethically feasible when robust and rigorous safeguards are established. Directed organ donation after euthanasia would fulfil the wish of the patient who is conscious, competent and able to provide first person consent, and it would be consistent with the same principles that permit directed donation for living donors. However, strict safeguards should be in place for the willing donor to protect this patient from any external pressure to request or continue a directed organ donation after euthanasia procedure.

## Data Availability

The original contributions presented in the study are included in the article/supplementary material, further inquiries can be directed to the corresponding author.
